# Update on CSF Biomarkers in Parkinson’s Disease

**DOI:** 10.3390/biom12020329

**Published:** 2022-02-18

**Authors:** Eun Hae Kwon, Sabrina Tennagels, Ralf Gold, Klaus Gerwert, Léon Beyer, Lars Tönges

**Affiliations:** 1 Department of Neurology, St. Josef-Hospital, Ruhr-University Bochum, D-44791 Bochum, Germany; eunhae.kwon@rub.de (E.H.K.); sabrina.tennagels@rub.de (S.T.); ralf.gold@rub.de (R.G.); 2 Center for Protein Diagnostics (ProDi), Ruhr University Bochum, D-44801 Bochum, Germany; klaus.gerwert@rub.de (K.G.); leon.beyer@rub.de (L.B.); 3 Faculty of Biology and Biotechnology, Department of Biophysics, Ruhr University Bochum, D-44801 Bochum, Germany

**Keywords:** cerebrospinal fluid, biomarkers, Parkinson’s disease, pathophysiology, α-synuclein

## Abstract

Progress in developing disease-modifying therapies in Parkinson’s disease (PD) can only be achieved through reliable objective markers that help to identify subjects at risk. This includes an early and accurate diagnosis as well as continuous monitoring of disease progression and therapy response. Although PD diagnosis still relies mainly on clinical features, encouragingly, advances in biomarker discovery have been made. The cerebrospinal fluid (CSF) is a biofluid of particular interest to study biomarkers since it is closest to the brain structures and therefore could serve as an ideal source to reflect ongoing pathologic processes. According to the key pathophysiological mechanisms, the CSF status of α-synuclein species, markers of amyloid and tau pathology, neurofilament light chain, lysosomal enzymes and markers of neuroinflammation provide promising preliminary results as candidate biomarkers. Untargeted approaches in the field of metabolomics provide insights into novel and interconnected biological pathways. Markers based on genetic forms of PD can contribute to identifying subgroups suitable for gene-targeted treatment strategies that might also be transferable to sporadic PD. Further validation analyses in large PD cohort studies will identify the CSF biomarker or biomarker combinations with the best value for clinical and research purposes.

## 1. Introduction

As one of the most common neurodegenerative disorders, Parkinson’s disease (PD) is also the fastest growing neurological disorder with regard to age-standardized rates of prevalence, disability and deaths [1]. Despite the relentless efforts and progress in unraveling the pathophysiological mechanisms of PD, a breakthrough in disease-modifying therapies is still lacking. The current PD diagnostic criteria mainly rely on the core motor symptoms—bradykinesia, rigidity and rest tremor. Even though these criteria are correctly applied, the rate of misdiagnosis is still up to 20% due to clinical overlap with parkinsonism of other etiologies [2]. Major differential diagnoses of PD include atypical parkinsonian syndromes (APSs) that share similar motor features of bradykinesia and rigor but are neuropathologically distinct disease entities. As with PD, multiple system atrophy (MSA) and dementia with Lewy bodies (DLB) belong to the spectrum of synucleinopathies, whereas progressive supranuclear palsy (PSP) and corticobasal syndrome (CBS) represent tauopathies. By the time of the first motor signs, at least 50% of nigral dopaminergic neurons are already lost [3], confounding the prospect to substantially alter the disease course. The asymptomatic preclinical and prodromal phases arising with the first non-motor and subtle motor signs provide an optimal window of therapeutic opportunity [4]. The possibility that early onset and progression of neurodegeneration could be accompanied by molecular changes measurable even before clinical onset constitutes the driving force of the biomarker search. Objective and reliable biomarkers are urgently needed, firstly, that identify PD in pre-motor stages and indicate susceptibility to the disease. Secondly, biomarkers should support the clinical diagnosis and define disease subtype and severity. Thirdly, biomarkers should reliably track disease progression and serve as meaningful endpoints for clinical trials to testify the impact on disease modification of an intervention. 

**Figure 1:**
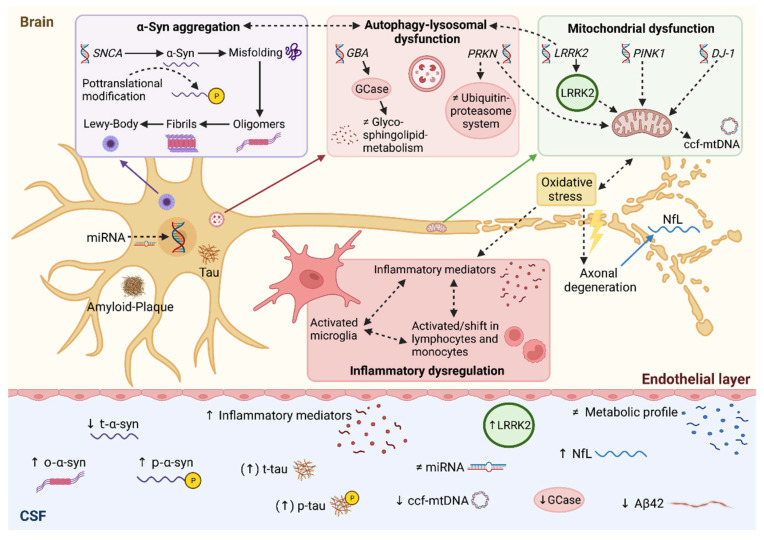
Synopsis of CSF biomarkers under investigation in Parkinson’s disease. Pathophysiological links between autophagy-lysosomal disruption, mitochondrial dysfunction and neuroinflammation leading to α-synuclein accumulation. Accordingly, molecular changes can be detected in CSF serving as candidate biomarkers in Parkinson’s disease. Solid lines represent direct relations, whereas dotted lines represent multi-step processes. ↑ increased; ↓ decreased; ≠ unaltered; Aβ42, amyloid beta peptide 1-42; α-syn, α-synuclein; ccf-mtDNA, circulating cell-free mitochondrial DNA; miRNA, microRNA; NfL, neurofilament light chain; o-α-syn, oligomeric α-synuclein; p-α-syn, phosphorylated α-synuclein; t-α-syn, total α-synuclein; p-tau, phosphorylated tau; t-tau, total tau protein. Created with BioRender.com (accessed on 8 January 2022).

Cerebrospinal fluid (CSF) represents the preferred source for biomarker discovery because of its direct contact with the extracellular space in the brain where an unrestricted bidirectional flow of molecules takes place between these compartments, secluded from the systemic circulation by the blood–brain barrier. However, only 20% of CSF proteins are brain-derived, while 80% are derived from filtration of the peripheral blood [5]. Nevertheless, compared to other peripheral fluids, these 20% brain-derived components have the greatest potential to truly reflect the state of the brain under pathological conditions. CSF is obtained by lumbar puncture, a procedure which is feasible in PD research participants, with a manageable rate of headache and lower back pain as adverse events [6]. Monitoring of disease progression and treatment response could be more challenging if repeated lumbar punctures would be required given the relative invasiveness of the procedure. Recent developments in CSF biomarker research in PD will be summarized in the following. Here, focus is directed to markers that reflect the pathophysiological mechanisms involved in PD including genetic aspects and examples of untargeted biomarker discovery approaches, regarding their usefulness and limitations (Figure 1). 

## 2. Alpha-Synuclein

Abnormal deposition of α-synuclein (α-syn) in the form of Lewy bodies represents the major neuropathological feature in PD associated with dopaminergic cell death. Numerous factors including genetic predisposition and post-translational modifications are considered to promote misfolding and aggregation of α-syn, leading to the subsequent formation of oligomers, amyloid-like fibrils and Lewy bodies [7]. 

Based on the central role of α-syn in PD pathogenesis, great attention has been paid to α-syn levels in CSF as a promising biomarker. Notably, the majority of studies consistently report lower CSF levels of total α-syn (t-α-syn) as compared to healthy controls [8,9,10], in contrast to the inconclusive findings in peripheral blood [11,12,13]. However, CSF t-α-syn levels vary greatly among studies, likely due to clinical heterogeneity and methodological differences that could compromise diagnostic accuracy. Indeed, a pooled sensitivity of 78–88% and a specificity of 40–57% for t-α-syn in CSF are still unsatisfactory to sufficiently discriminate PD from controls [14]. Differential diagnosis cannot be supported by t-α-syn in CSF alone, since MSA and DLB, and even tauopathies such as PSP and CBS, also show reduced levels [15,16]. The significance of CSF α-syn in Alzheimer’s disease (AD) is still unclear despite Lewy body co-pathology in AD. Correlations of the CSF α-syn level with AD markers have been reported [17,18]. Since Lewy bodies have also been found in neurologically asymptomatic elderly individuals [19], it would be important to target disease-specific forms of α-syn. Longitudinal observations of t-α-syn dynamics in CSF revealed discrepant findings showing an increase in t-α-syn throughout the disease course [20,21], but also a decrease without correlation with disease progression [22]. Divergence of α-syn concentrations in CSF could additionally result from the fact that α-syn is deposited in Lewy bodies but also released from degenerating synapses [23]. 

Apart from the total level of α-syn, other species of α-syn can be measured in CSF. Post-translationally modified phosphorylated α-syn (p-α-syn) levels in CSF were increased in PD patients compared to controls [24] and decreased during the disease course [20], indicating disease progression. Interestingly, it has been shown that p-α-syn could be detected by ultrasensitive immunoassays only in plasma, but not in CSF [25]. This finding underlines the instability of post-translationally modified species and the possible influence of matrix effects leading to interference between proteins or other constituents present in the CSF and the assay used for detection. For improving the diagnostic utility, CSF levels of oligomeric α-syn (o-α-syn) and the ratio of o-α-syn/t-α-syn have been investigated and found to be elevated in PD patients [20,26,27]. CSF levels of o-α-syn showed a longitudinal increase, and the change in o-α-syn/t-α-syn showed an association with motor deterioration, particularly in the postural-instability and gait-difficulty dominant PD subgroup [20]. 

In recent years, two ultrasensitive protein amplification assays, Protein Misfolding Cyclic Amplification (PMCA) and the Real-Time Quaking-Induced Conversion (RT-QuIC), have been introduced to detect aggregated and misfolded α-syn in CSF, yielding high diagnostic accuracy (AUC 0.93 for PMCA and 0.89 for RT-QuIC) in distinguishing PD from controls [28]. A high sensitivity (95.3%) and specificity (98%) of α-syn seeding activity were demonstrated by applying RT-QuIC to accurately discriminate synucleinopathies [29]. Excellent separation of MSA from PD/DLB subjects could be achieved by PMCA-based o-α-syn analysis [29,30]. Interestingly, the PMCA method could also detect specific α-syn strains forming different conformational aggregates that could reliably distinguish between MSA and PD [31]. Further efforts are needed to confirm the ability of different α-syn species or conformational states to validate the clinical value in terms of precise PD diagnostics. The robustness of immunoassays or aggregation assays relies on the quality of standardized antigens for quantification. In addition, blood contamination constitutes a considerable challenge for accurate quantification of the α-syn level in CSF [32].

## 3. Amyloid-Beta and Tau Protein 

Besides the PD-defining synuclein pathology, other age-related neurodegenerative pathologies can coexist in PD brains including amyloid plaques and tau-containing neurofibrillary tangles that are classical features of AD [33,34,35]. In AD, the diagnostic usefulness of CSF biomarkers has already been proven, which provides a great impetus to implement CSF biomarkers in other neurodegenerative disorders such as PD. Amyloid-β (Aβ) and tau protein can interact with α-syn, thus promoting their mutual accumulation that contributes to the accelerated cognitive decline in PD [36]. Analogous to AD, several studies reported that a lower CSF level of amyloid-beta_1-42_ (Aβ42) at baseline compared to controls could predict cognitive impairment possibly reflecting amyloid pathology [27,37,38,39]. The reduction in the CSF Aβ42 level in LBD, AD and PD with dementia (PDD) tends to be more pronounced compared to PD, but differential diagnosis remains difficult solely based on Aβ42 levels and requires the combination of other markers [40,41]. In the Parkinson’s Progression Markers Initiative (PPMI) cohort, early PD subjects were followed for up to three years [42]. Aβ42 in CSF showed a greater decrease during the disease course, and a lower Aβ42 level at baseline predicted a modest decline not only in cognitive but also in autonomic and motor functions in early PD. Interestingly, a low baseline CSF Aβ42 level was also able to predict the progression of dopa-resistant gait impairments in PD [43]. By normalizing the Aβ42 concentration to amyloid-beta_1-40_ (Aβ40), the most abundant form of Aβ peptides, the Aβ42/Ab40 ratio can correct for interindividual differences and should hence be included in future PD investigations. 

Although total tau (t-tau) and phosphorylated tau (p-tau) are implicated in tau pathology and cognitive dysfunction in AD, the role of CSF tau species in PD has not been clarified yet. There are mixed results showing reduced [44], similar [45] and increased CSF levels of t-tau and p-tau, particularly in PD with manifest dementia [46]. With regard to differential diagnosis of MSA, higher t-tau levels have been observed in MSA compared to PD [47,48]. The increase in t-tau and p-tau in CSF could mirror unspecific events of neuronal damage caused by stroke or viral encephalitis and is even higher in patients with Creutzfeld–Jakob disease [49]. However, even for tauopathies other than AD (frontotemporal dementia (FTD) and PSP), CSF tau concentrations are not significantly different from healthy controls [50]. Lower secretion of tau proteins in the extracellular space for these tauopathies and alternative disease-specific tau processing that could escape detection from available assays have been discussed. Therefore, the question whether an increase in tau levels in APS could reflect a more rapid progression compared to PD needs to be further elucidated. At least in combination with Aβ42, increased t-tau and p-tau levels in CSF allowed the prediction of subsequent decline in cognitive tasks involving both memory and executive functions [51]. 

## 4. Neurofilament Light Chain 

Neurofilament light chain (NfL) is a subunit of neurofilaments that is exclusively expressed in neurons. It is the main structural component of large, myelinated axons. Following axonal damage and membrane disruption, neuronal signals are interrupted and NfL is released in the interstitial space [52]. As a general marker of axonal injury, NfL has been extensively studied in the field of neurodegeneration. Higher NfL levels have been reported in the CSF of PSP and MSA patients in comparison to PD patients, consistent with the more aggressive neurodegeneration in these disease entities [53,54]. A comprehensive meta-analysis exhibited no differences in the mean CSF NfL values of PD and PDD/DLB patients, but increased levels in MSA, PSP and CBS patients [55]. NfL levels in CSF were investigated in a longitudinal cohort of de novo PD patients (DeNoPa), demonstrating the highest levels in other neurodegenerative diseases including MSA and DLB compared to PD and the lowest levels in controls [56]. Higher NfL levels in CSF could perfectly separate MSA patients from controls, and higher cut-off values enabled excellent discrimination of MSA from PD and DLB (97% sensitivity, 90% specificity) since NfL was not elevated in the CSF of most PD and DLB cases [30]. A recent study demonstrated the highest CSF NfL level in PD subjects with cognitive impairment and a moderately elevated level in PD subjects with normal cognitive function compared to the control group [57]. Although CSF NfL correlated with motor and cognitive impairment, the conversion to cognitive impairment could not be predicted by the baseline NfL level. Moreover, it has been shown that NfL correlates with age, and lacking age-specific reference values could hamper the accurate distinction of PD from elderly controls [57,58]. These results underline the potential of NfL as a candidate for a biomarker panel in combination with other markers, but there is also great overlap across the different studies and disease groups and a critical correlation with the aging of the patients. 

## 5. Lysosomal Biomarkers

The autophagy–lysosomal pathway is a key route for the intracellular degradation of proteins. A disturbed autophagy–lysosomal system can cause a reduced degradation of α-syn, thus enhancing α-syn accumulation in PD [59]. This hypothesis is strengthened by the identification of mutations in the GBA gene, encoding the lysosomal enzyme glucocerebrosidase (GCase), as the most common genetic risk factor for PD [60,61]. It can be assumed that reduced GCase activity elevates the α-syn level by stabilizing toxic soluble oligomeric α-syn which, in turn, leads to decreased lysosomal activity in a bidirectional pathogenic loop [62]. Not only GBA but also an excessive burden of 54 other lysosomal storage disorder gene variants has been linked to PD [63]. Following this pathophysiological pathway, markers of lysosomal metabolism have been investigated in CSF as candidate biomarkers for PD. 

GCase is an enzyme that hydrolyzes glucosylceramide (GlcCer) and glucosylsphingosine (GlcSph) into glucose and ceramide or sphingosine, respectively [64]. CSF GCase activity has been shown to be significantly reduced in PD as well as in DLB compared to controls [65,66,67,68,69,70]. In PD patients carrying a GBA mutation (GBA-PD), CSF GCase activity was significantly lower compared to sporadic PD (sPD), whereas GBA-PD with severe mutations showed the lowest enzyme activity, indicating an accelerated pathological condition in GBA mutations [71]. However, other studies reported a reduced GCase activity in the CSF of PD patients irrespective of GBA mutations [67,68]. This could be partially explained by the fact that aging also leads to a progressive decline in CGase activity [72]. Activities of other lysosomal hydrolases, such as β-hexosaminidase and β-galactosidase, were also found reduced in the CSF of GBA-PD and sPD compared to controls [67,70]. Regarding downstream metabolites, a significant elevation in ceramide species has been reported in PD and other Lewy body spectrum disorders independent of GBA mutation [64,71]. Kurzawa-Akanbi and colleagues postulated an upregulation of ceramides in response to cell stress [64]. Ceramides were found heavily loaded in extracellular vesicles together with neurodegeneration-linked proteins including α-syn and tau, possibly mediating α-syn aggregation by interaction of these molecules. A moderate diagnostic accuracy of lysosomal enzymes could only be yielded in combination with other markers. A combination of GCase activtity, the o-/t-α-syn ratio and age showed the best performance in discriminating PD from controls independent of GBA mutation status (sensitivity of 82% and specificity of 71%) [68]. The diagnostic accuracy could be further improved by using a combined lysosomal enzyme profile (β-glucocerebrosidase, cathepsin D and β-hexosaminidase) [67]. 

Some studies even suggest a prognostic value of lysosomal markers. In the longitudinal observation of the PPMI cohort including GBA-PD and sPD, the glucosylceramide fraction was increased, whereas the sphingomyelin fraction was reduced in the CSF of GBA-PD patients compared to controls [73]. However, a higher ratio of glucosylceramide to sphingomyelin significantly associated with an accelerated cognitive decline in sPD compared to the sPD subjects with a lower ratio. Importantly, this finding indicates that genetically derived findings could be transferred to sPD and used for stratification in clinical trials. Another study reported that reduced lysosomal enzymes GCase and cathepsin D significantly associated with more advanced motor stages in Hoehn and Yahr (H&Y), while lower cathepsin D and β-hexosaminidase activities significantly associated with worse cognitive performance [67]. 

In conclusion, lysosomal biomarkers are promising since lysosomal metabolism appears to be crucially involved in the pathophysiology of PD and significant alterations could be measured. While lysosomal progression markers need further investigation, the diagnostic accuracy can be enhanced by considering PD-related pathologies in combination, such as CSF levels of α-syn or AD core biomarkers [67,68]. Therefore, the potential lysosomal biomarkers for diagnosis should be included in a robust biomarker panel covering the different pathological pathways of PD.

## 6. Inflammatory Biomarkers

Neuroinflammation plays a major role in the pathology of PD, since α-syn triggers the activation of the innate and adaptive immune systems [74]. Humoral and cellular components of the immune system have been investigated in CSF as candidate biomarkers for PD. Phenotyping of CSF immune cells by multiparameter flow cytometry in PD patients revealed a shift in cell proportions from classical monocytes (CD14+/CD16−) to non-classical monocytes (CD14+/CD16+) in PD patients compared to controls, which was not the case in the peripheral blood [75]. In PD patients, the fraction of activated T lymphocytes was found to be increased, whereas the absolute numbers of these cell populations were not significantly altered [75]. Studies on the humoral inflammatory profile highlight two promising inflammatory markers, namely, monocyte chemoattractant protein-1 (MCP-1) and YKL-40 (chitinase-3-like protein 1). MCP-1 is a chemokine that is involved in the recruitment of monocytes and spreading of inflammation. Elevated MCP-1 levels were detected in the CSF of PD [75] as well as MSA patients in comparison to controls [76]. On the contrary, other studies reported comparable MCP-1 levels in the CSF among PD, MSA and controls [77,78,79,80,81,82]. These discrepancies might be explained by clinical heterogeneity, diagnostic uncertainties and different assays used. Despite the diagnostic insufficiency, a positive correlation was found with motor progression (H&Y) [77,78] and even the severity of depression as a non-motor symptom [82]. YKL-40 is a glycoprotein primarily expressed in microglia and astrocytes. There are inconsistent results on the trend of CSF levels and differentiating biomarker potential among PD, APS and controls [76,81,83,84,85,86]. However, an increase in YKL-40 in CSF over time in PD correlated significantly with faster cognitive decline [21]. Further studies support its potential to predict cognition biomarkers in AD [81] and PD [83]. 

Elevated CSF levels of C-reactive protein (CRP), an acute phase protein and a commonly applied inflammatory marker, showed a significant correlation with the severity of motor (H&Y, UPDRS III) and non-motor (cognition, depression and fatigue) symptoms in PD and APS [82,84]. Further studies detected significantly higher CRP levels in PDD and MSA compared with non-demented PD and controls [82,84], supporting its predictive value for cognitive decline [87]. Several other potential inflammatory markers have been suggested including TNF-α [75,88,89,90], fractalkine [91,92], MIP1α (CCL3) [92], IL-1β [89], IL-2 and -6 [75,84,89], IL-8 [84], TGF-β1 and IFN-γ [89,93,94,95]. Each marker on its own can only cover a restricted biological domain and fails to reach statistical significance. Accordingly, combining different markers into robust large panels can lead to higher diagnostic sensitivity [78,88,89,90,93]. For instance, CRP and a cytokine set (TNF-α, IL-1β, IFN-γ) were able to distinguish PD from MSA [89]. The p-tau/α-syn ratio combined with TNF-α could separate PD patients from controls (AUC > 0.9) [90]. Therefore, inflammatory biomarkers should be included in comprehensive panels as they may particularly reflect motor and non-motor PD progression, particularly in more aggressive PD forms [94].

## 7. Metabolomics

A non-hypothesis-driven approach for the discovery of new biomarkers can be achieved by untargeted metabolomics [96]. Metabolomics has attracted attention in recent years, revealing multiple novel metabolic pathways linked to the pathogenesis of PD. Several studies demonstrated altered metabolic profiles in PD, PD subgroups, APS and other neurological conditions [97,98,99,100,101,102,103]. By utilizing machine learning algorithms, 14 CSF metabolites were identified that enabled distinguishing early PD from controls with high accuracy [104]. These metabolites indicated alterations of the amino acid metabolism in PD, although the exact mechanisms associated with PD need to be clarified. Another cohort with early PD patients (DeNoPa) reported a significant decrease in dehydroascorbic acid CSF levels and a significant increase in fructose, mannose and threonic acid CSF levels—molecules that are involved in the antioxidative stress response, glycation and inflammation—compared to controls [105]. Moreover, a link between altered metabolic profiles and the tricarboxylic acid cycle has been observed, which are implicated in mitochondrial dysfunction and increased oxidative stress [106]. By using liquid chromatography–tandem mass spectrometry, a recently published study detected increased CSF levels of intermediates of the proline metabolic pathway in PD and APS subjects that could discriminate them from controls but not among parkinsonian syndromes [97]. 

Concerning PD medication-related sequelae, dysregulations in bile acid biosynthesis and glycosphingolipid/glycerophospholipid metabolism were able to distinguish PD patients suffering levodopa-induced dyskinesia (LID) from PD without LID and controls, pointing towards a further association between dysregulated lipid metabolism and neuroinflammation [98]. Intriguingly, an altered glycosphingolipid metabolic pathway was strongly associated with the severity of dyskinetic movements. In terms of PD progression markers, CSF concentrations of the main dopamine metabolite and end product of dopamine catabolism, homovanillate, showed only a slight change over time and a weak correlation with worsening of disease severity, lacking sufficiency to reflect disease progression [107]. To sum up, these findings illustrate the complexity of the multiple metabolic pathways involved in the pathogenesis of PD. Further studies are needed to better understand the pathophysiological context and validate these promising metabolites. 

## 8. Genetic Perspective

Even though only a minority of PD cases (5–10%) are caused by monogenic mutations, understanding the genetic basis has provided fundamental insights into PD pathogenesis and led to the development of gene-targeted treatment strategies [108,109]. Genetic PD includes autosomal-dominant (SNCA, LRRK2) and autosomal-recessive forms (Parkin, PINK1, DJ-1) and the most common genetic risk factor GBA. A genetic trait is considered stable and indicates the predisposition to develop a disease, albeit with incomplete and variable penetrance [110]. In addition to the gene mutation itself, gene products at the transcriptional and post-transcriptional levels in CSF could serve as markers that possibly reflect the pathophysiological processes underlying PD. 

The biomarker potential of α-syn (encoded by SNCA) and GCase (encoded by GBA) has been extensively discussed in the previous sections. Although SNCA mutations are very rare, the finding of α-syn containing Lewy bodies establishes a pivotal link between genetic and sporadic forms of PD [111]. GBA mutations represent a risk factor for PD with a reduced penetrance, and about 9.1% of GBA mutation carriers will develop PD [112]. Ambroxol is a chaperone for GCase and discussed as a novel neuroprotective agent. In acellular CSF, the inhibitory effect of ambroxol can be measured by decreased GCase activity, whereas in tissue, CGase activity is supposed to increase, and target engagement can be determined by upregulation of CSF GCase protein levels [113]. Among LRRK2 mutations, G2019S is the most frequent variant, causing monogenic PD with age- and population-dependent incomplete penetrance [114,115]. LRRK2 encodes a multifunctional protein including a kinase domain and exerts its physiological role in cytoskeletal maintenance, mitochondrial function and autophagy [116]. Since most pathogenic variants lead to increased kinase activity, pharmacological LRRK2 inhibition has been proposed as a counteractive therapeutic strategy. LRRK2 was first detected in exosomes purified from CSF [117]. A more reliable parameter to quantify LRRK2 kinase activity is the measurement of the level of autophosphorylation at pS1292 [118]. Compared to pS1292-LRRK2 levels in urinary exosomes, CSF levels were much higher (about 5-fold) but failed to discriminate LRRK2 mutation carriers/PD patients from non-carriers/controls, possibly due to saturation effects. By using an improved LRRK2 monoclonal antibody technique, absolute quantification of the LRRK2 protein has revealed elevated CSF LRRK2 levels in G2019S-PD compared to sPD and non-manifesting G2019 carriers [119]. Autosomal-recessive forms of PD with typical early onset are most frequently linked to mutations in Parkin, followed by PINK1 and very rarely DJ-1 [108]. These genes participate in mitochondrial quality control, disruption of which is thought to significantly contribute to PD pathogenesis [120,121]. PINK1 is a mitochondrial kinase that phosphorylates Parkin, an E3 ubiquitin ligase, to eliminate damaged mitochondria. DJ-1 is also involved in mitochondrial regulation and antioxidative stress mechanisms. In response to oxidative stress, circulating cell-free mitochondrial DNA (ccf-mtDNA) is released from cells. Paradoxically, a lower ccf-mtDNA level was reported in the CSF of PD patients, which could be explained by shutting down energy production prior to cell death [122]. The CSF ccf-mtDNA level could be influenced by medication as demonstrated by the inverse correlation with treatment [123]. Results regarding DJ-1 are inconclusive since increased and decreased CSF levels have been reported in PD [124]. Therefore, it remains disputable whether DJ-1 in CSF is able to differentiate PD from APS or controls [47,125].

MicroRNAs (miRNAs) are small noncoding RNAs that are involved in the post-transcriptional regulation of gene expression through inhibition of translation and degradation of target mRNAs [126]. They can be detected cell-free or within extracellular vesicles, particularly exosomes, and remain stable in various body fluids such as CSF [127]. Distinct miRNA signatures have been found in the CSF of early [128] as well as more advanced PD stages [129,130,131], differentiating them from controls and APS [130,132]. However, since standardized methodological and normalization approaches are still missing, inconsistencies between miRNA studies and lack of reproducibility hamper miRNAs from becoming more widely used as biomarkers [133].

## 9. Conclusions 

The research of CSF biomarkers has deepened our understanding of the biological and molecular processes occurring in the brain. Table 1 summarizes candidate CSF biomarkers for PD under current investigation. In view of the pathophysiological evidence, α-syn species have the strongest rationale of use and should constitute the basis of composite biomarker panels. Fortunately, novel techniques such as PMCA and RT-QuIC have improved the detection of α-syn aggregates. CSF Aβ42 has proved its prognostic use for cognitive impairment in PD. Other biomarkers related to axonal damage (tau proteins and NfL) are not specific for PD diagnosis but can help assess PD progression. Moreover, multiple novel candidate biomarkers have been identified within known (lysosomal, inflammatory, mitochondrial dysfunction, LRRK2) and novel biological pathways associated with PD (metabolomics). Given the complexity and intricate interplay of different pathophysiological mechanisms, applying a panel of markers reflecting different aspects of disease-related pathways simultaneously would be most promising. In order to optimize the utility of CSF biomarkers, analytical validation is needed by establishing standardization of techniques (including assays for measurement, sample collection and handling procedures) across different laboratories that can ensure the reproducibility of results and the generation of relatable cut-off and calibration values. Further validation analyses in large PD cohort studies will identify the CSF biomarker or biomarker combinations with the best value for clinical and research purposes. Importantly, confounding factors such as age should be excluded by adjusting models accordingly. Even though laborious and invasive, the number of longitudinal CSF studies needs to be expanded for prognostic assessment. Large multicentric longitudinal biomarker studies including PD at the very early stages (i.e., pre-motor PD) will allow identifying relevant molecular changes for early diagnostic accuracy. Furthermore, the prognostic value of certain markers needs to be ascertained for their use as indicators of disease progression and treatment-associated changes that are imperative for proving the effectiveness of novel disease-modifying therapeutics. 

## Figures and Tables

**Table 1:** Overview of CSF biomarkers according to key pathologic mechanisms involved in PD with diagnostic and prognostic relevance indicating disease severity and progression.

Pathomechanism	CSF Biomarker	Differential-/Diagnosis	Biomarker Changes in Advanced Disease
α-syn misfolding and aggregation	t-α-syn	↓ PD/APS vs. HC	No certain correlation with disease progression
	p-α-syn	↑ PD vs. HC	↓ over disease course
	o-α-syn	↑ PD vs. HC	↑ over disease course ↑ o-/t-α-syn ratio correlates with motor progression
	α-syn aggregates	↑ PD/MSA/DLB vs. HC	
Amyloidosis	Aβ42	↓ DLB/AD/PDD vs. PD/HC	↓ predicts earlier cognitive decline
Tauopathy	t-tau	↑ MSA vs. PD	↑ t-/p-tau plus Aβ42 predicts cognitive decline
	p-tau	Inconclusive	
Axonal damage	NfL	↑ APS > PD	↑ correlates with motor and cognitive impairment
Autophagy–lysosomal pathway dysfunction	GCase	↓ sPD/GBA-PD/DLB vs. HC	↓ in more advanced motor stages
	cathepsin D, β-hexosaminidase	↓ PD vs. HC	↓ correlates with worse cognitive performance
	GlcCer, SM	↓ GBA-PD vs. HC	↑ GlcCer/SM ratio correlates with accelerated cognitive decline in sPD
Neuroinflammation	immune cell composition	Shift in PD vs. HC	
	MCP-1	↑ PD/MSA vs. HC	↑ correlates with motor progression and depression
	YKL-40	Inconclusive	↑ correlates with faster cognitive decline
	CRP	↑ PDD/MSA vs. PD/HC	↑ correlates with motor and non-motor symptoms
Altered metabolic pathways	threonic acid, mannose, fructose	↑ PD vs. HC	
	proline metabolites	↑ PD/APS vs. HC	
	glycosphingolipid metabolism	PD with LID vs. PD without LID	Correlation with severity of dyskinesia
↑ LRRK2 kinase activity	pS1292-LRRK2	LRRK2-PD = sPD/HC	
	LRRK2	↑ LRRK2-PD vs. sPD	
Mitochondrial dysfunction (PINK1/Parkin/DJ1)	Ccf-mtDNA	↓ PD vs. HC	↓ correlates with ↑PD medication
	DJ-1	Inconclusive	
Regulation of gene expression	miRNA	Altered profile in PD vs. HC	

Abbreviations: o-α-syn, oligomeric α-synuclein; p-α-syn, phosphorylated α-synuclein; t-α-syn, total α-synuclein; PD, Parkinson’s disease; APS, atypical parkinsonian syndromes; HC, healthy controls; MSA, multiple system atrophy; DLB, dementia with Lewy body; PDD, PD dementia; GBA-PD, PD linked to mutation in the glucocerebrosidase gene (GBA); sPD, sporadic PD; LID, levodopa-induced dyskinesia; Aβ42, amyloid beta peptide 1-42; NfL, neurofilament light chain; GCase, glucocerebrosidase; GlcCer, glucosylceramide; SM, sphingomyelin; MCP-1, monocyte chemoattractant protein-1; YKL-40, Chitinase 3-like 1; CRP, C-reactive protein; ccf-mtDNA, circulating cell-free mitochondrial DNA; miRNA, microRNA; ↑, increased levels; ↓, decreased levels.
